# Disruption of Neuromuscular Junction Following Spinal Cord Injury and Motor Neuron Diseases

**DOI:** 10.3390/ijms25063520

**Published:** 2024-03-20

**Authors:** Colin Nemeth, Naren L. Banik, Azizul Haque

**Affiliations:** 1 Department of Microbiology and Immunology, Medical University of South Carolina, 173 Ashley Avenue, Charleston, SC 29425, USA; cnemeth@villanova.edu (C.N.); baniknl@musc.edu (N.L.B.); 2 Department of Neurosurgery, Medical University of South Carolina, 96 Jonathan Lucas Street, Charleston, SC 29425, USA; 3 Ralph H. Johnson Veterans Administration Medical Center, 109 Bee Street, Charleston, SC 29401, USA

**Keywords:** spinal cord injury, inflammation, axonal damage, skeletal muscle, myasthenia gravis, neuromuscular junction

## Abstract

The neuromuscular junction (NMJ) is a crucial structure that connects the cholinergic motor neurons to the muscle fibers and allows for muscle contraction and movement. Despite the interruption of the supraspinal pathways that occurs in spinal cord injury (SCI), the NMJ, innervated by motor neurons below the injury site, has been found to remain intact. This highlights the importance of studying the NMJ in rodent models of various nervous system disorders, such as amyotrophic lateral sclerosis (ALS), Charcot–Marie–Tooth disease (CMT), spinal muscular atrophy (SMA), and spinal and bulbar muscular atrophy (SBMA). The NMJ is also involved in myasthenic disorders, such as myasthenia gravis (MG), and is vulnerable to neurotoxin damage. Thus, it is important to analyze the integrity of the NMJ in rodent models during the early stages of the disease, as this may allow for a better understanding of the condition and potential treatment options. The spinal cord also plays a crucial role in the functioning of the NMJ, as the junction relays information from the spinal cord to the muscle fibers, and the integrity of the NMJ could be disrupted by SCI. Therefore, it is vital to study SCI and muscle function when studying NMJ disorders. This review discusses the formation and function of the NMJ after SCI and potential interventions that may reverse or improve NMJ dysfunction, such as exercise, nutrition, and trophic factors.

## 1. Introduction

The neuromuscular junction (NMJ) is a basic synapse model between motor nerve terminals and muscle fibers [1,2,3]. Although the NMJ is presented as a simple model, it is a complicated set of mechanisms involving many parameters in the delicate structure. This junction is responsible for all muscle movement, relaying information from the nervous system to the muscles. Spinal cord injury (SCI) refers to harm sustained by the compact cluster of cells and nerves responsible for transmitting signals between the brain and the rest of the body [4]. This vital conduit, known as the spinal cord, stretches from the base of the brain to the lower back. SCI can injure the NMJ [4,5]. Axonal damage, a characteristic feature of spinal cord injury (SCI), profoundly disrupts the communication between the central nervous system (CNS) and the peripheral nervous system (PNS) [6,7]. Primary mechanical trauma and secondary processes like inflammation and oxidative stress contribute to this disruption. Resulting in sensory, motor, and autonomic deficits, axonal injury also compromises trophic support, exacerbating neuronal loss and functional impairment. Understanding these dynamics is crucial in developing targeted therapies to restore neural connectivity and improve outcomes for SCI patients. This disruption extends to the NMJ, the nexus responsible for translating neural signals into muscle contractions [8,9,10]. The consequences are far-reaching, with potential impacts on motor function, muscle strength, and overall mobility. If there were any injury or degradation to this essential junction, many motor functions would be impaired, and there could be possible cell death or muscle atrophy.

The NMJ has three major sections: a presynaptic motor neuron terminal, a synaptic cleft, and a postsynaptic muscle membrane/motor endplate [11,12]. The presynaptic motor neuron terminal terminates a neuron’s distal axon. The primary function of the presynaptic terminal is to fuse neurotransmitter-filled vesicles into the neural cell membrane. The synaptic cleft is the extracellular matrix between the presynaptic and postsynaptic terminals [13,14]. The terminal phase of the neuromuscular junction involves the postsynaptic muscle membrane. This membrane exhibits a series of invaginations, thereby augmenting its surface area to facilitate the enhanced uptake of neurotransmitters. The junction is simple in understanding its parts but very complex in its function. As the NMJ matures during the lifespan, there are apparent changes in its morphology, such as enlargements and muscle invaginations forming. However, in the adult NMJ, many hidden mechanisms prove that any injury or disease affecting its stability will affect the greater organism [15,16,17].

It is an essential target for various nervous system disorders, such as amyotrophic lateral sclerosis (ALS), Charcot–Marie–Tooth disease (CMT), and SMA [18,19,20]. Visualizing and analyzing the NMJ in rodent models of these disorders is crucial, particularly during the early stages of the disease. The NMJ is also involved in myasthenic disorders such as myasthenia gravis (MG) and is vulnerable to neurotoxins [21,22,23]. Additionally, it is known to degrade before axon loss occurs in response to axotomy and is a common feature of aging. The NMJ has been widely studied in mice and rats due to the availability of their tissue for imaging techniques such as immunofluorescence and electron microscopy [3,24,25]. The NMJ undergoes significant changes in its morphology during the early postnatal period. The postsynaptic endplate of the NMJ changes from a simple, circular shape to a more complex structure as the motor neuron matures [15,16,17]. Lower motor neuron degeneration is a characteristic of several neuromuscular disorders, and the NMJ is often an early target in these disorders in rodent models. It is, therefore, important to accurately assess the level of denervation at the NMJ. Fully denervated synapses can be easily identified, but determining whether the NMJ is fully innervated or partially denervated can be more subjective [24].

Specifically, spinal cord atrophy conditions or injury may significantly affect motor neurons and their processes, branching from the spinal cord to the skeletal muscle. SCI is caused by automobile accidents, gunshots, wounds, falls, and others; these have devastating effects and may lead to paralysis. Depending upon the severity, very few people that undergo SCI recover fully and regain function. However, some neurodegenerative diseases and conditions may affect the longevity of the spinal cord and the nervous system’s health, along with traditional aging. Regardless of its cause, SCI can induce life-threatening or permanent disabilities because CNS neurons exhibit minimal regeneration [26,27,28,29]. Understanding the interplay between SCI and NMJ disorders is crucial due to the potential adverse effects on motor function, muscle atrophy, and long-term disability. The NMJ, although seemingly simple in its structure, plays a pivotal role in ensuring seamless communication between the nervous system and skeletal muscle [3,15,17]. Any disruption to this communication has the potential to significantly compromise an individual’s quality of life following SCI. Because of this and other complexities in CNS injuries, the current therapeutic options addressing SCI and its impact on the NMJ are limited. While rehabilitation and physical therapy aim to mitigate the functional consequences [2,30], there is a notable gap in strategies specifically targeting the repair and restoration of the NMJ [31,32]. This gap underscores the need for a comprehensive review that not only elucidates the existing challenges but also explores potential therapeutic options to address this critical void in the field.

The movement of animals, including humans, is an integral part of their lives. The interaction between the NMJ’s CNS and the skeletal muscles controls this movement [8,15]. At this site, electrical activity in the motor neuron causes the release of a chemical neurotransmitter that activates the contraction of nearby muscle cells. Any disruptions to this process can lead to muscle weakness or paralysis and negatively impact an animal’s quality of life. Many human conditions caused by mutations in over 30 genes involve impaired neuromuscular transmission [15]. These conditions often have limited treatment options. To improve the treatment of these conditions, it is crucial to understand the factors that affect NMJ function. These factors include the functioning of the proteins and molecular complexes involved in transmitter release and action and the NMJ’s structural features. While much is known about the molecular basis of transmitter release and action, the determinants of the NMJ’s structure are not well understood. This review will focus on SCI that may negatively affect the NMJ at the skeletal muscle junctions and possible treatments to repair the NMJ. It is necessary to juxtapose SCI and NMJ functionality because the two have significantly intense effects on the majority of patients affected.

## 2. NMJ Morphology

### 2.1. Structure of the NMJ

The cells in the central part of the body that control the movement of the skeletal muscles, called motor neurons, send out long, unbranched axons to the muscles. These axons then branch extensively and make contact with many muscle fibers [16,33]. The NMJ is a specialized synapse that forms between the lower motor neurons and skeletal muscle fibers. It is an essential component of the nervous system that allows communication between these two types of cells and enables muscle movement [24]. The NMJ can be divided into three main sections for ease of understanding: the part of the nerve that releases the transmitter (nerve terminal), the space between these two parts (synaptic cleft), and the part of the muscle that receives the transmitter (motor endplate) [34,35].

The nerve terminal is part of a neuron located at the end of an axon. It releases neurotransmitters when stimulated by an electrical signal traveling through the axon. This specialized region is separate from the main body of the neuron, or soma [36]. When a myelinated motor neuron reaches its target muscle, it sheds its myelin sheath [37,38]. It forms a network of around 100–200 branching nerve endings, also known as nerve terminals or terminal boutons. The rapid fusion of small vesicles called synaptic vesicles (SVs) at the terminals of neurons is essential in transmitting signals between cells [39]. It is thought that the organized cytomatrix, or the network of proteins within cells, plays a role in the regulated movement of SVs. However, the specifics of how the cytomatrix connects SVs and keeps them near the active zones where they are released, and how it organizes docked SVs at the release sites, need to be fully understood.

The space between the terminals of two cells, enclosed by adjacent cell membranes, is referred to as the synaptic cleft [40,41,42]. Different types of synapses with varying molecular compositions are used throughout various neural networks and pathways. These synapses allow for diverse connectivity patterns and functions. It has also been discovered that the space within the synaptic cleft plays a significant role in the structure and function of the synapse [43]. The presynaptic neurotransmitter acetylcholine (ACh) is released at the NMJ and interacts with nicotinic ACh receptors on the motor endplate. The synaptic cleft of the NMJ contains the enzyme acetylcholinesterase (AChE), which breaks down the released ACh to prevent its prolonged effect on the postsynaptic receptors [34,40].

The motor endplate is the part of the NMJ that receives signals from nerve endings. It is a thickened part of the muscle cell membrane that is folded to create small indentations called junctional folds [34]. These folds have a high concentration of nicotinic ACh receptors, channels in the cell membrane that ACh activates [44]. When ACh binds to these receptors, the channels open and allow positively charged sodium ions to flow into the muscle cell [34,45]. This triggers an electrical signal, called an action potential, that spreads across the muscle cell membrane and causes the muscle to contract. The terminal nerve endings do not directly contact the motor endplate but rather fit into the junctional folds. The motor endplate is a specific region of the sarcolemma that quickly and consistently reacts to the release of neurotransmitters from the nearby nerve terminal. For efficient neuromuscular transmission, the proper development and organization of the NMJ is essential [33,46,47].

### 2.2. Altered Structure of NMJ and Damage

The NMJ is vulnerable to damage in a variety of nervous system disorders, including amyotrophic lateral sclerosis (ALS), Charcot–Marie–Tooth disease (CMT), spinal and bulbar muscular atrophy (SBMA), and spinal muscular atrophy (SMA) [48,49,50,51]. These conditions can lead to NMJ degradation or dysfunction, which could impair muscle function and mobility. It is important to see and accurately measure the NMJ in rodents with neurological conditions, especially in the early stages of the disease [24]. This allows for a better understanding of the condition and potential treatment options to deal with NMJ dysfunction.

The mammalian NMJ is a peripheral synapse that functionally couples the lower motor neurons to skeletal muscle fibers [15,16]. Formed during prenatal development, the highly specialized NMJ efficiently transfers information from a presynaptic motor nerve to a postsynaptic muscle fiber [15,52], permitting rapid muscle contraction in response to neuronal stimulation. It is formed during fetal development and is a specialized structure that allows for the efficient transmission of information from the motor nerves to the muscle fibers [24]. This enables the rapid contraction of muscles in response to neuronal signals, which is essential for movement and other functions. The NMJ is a peripheral synapse located outside the CNS and is specifically designed to facilitate the transfer of information between the presynaptic motor nerve and the postsynaptic muscle fiber [53,54,55]. The NMJ is highly specialized and finely tuned to enable fast and efficient communication between these two cell types. Due to its relatively large size, experimental tractability, and simplicity, the NMJ has been used as a model for many years to better understand the general principles of synaptic establishment, structure, development, and function [13,56]. Little is known about the mechanism of presynaptic assembly, although a recent study showed the important roles of motoneuron Wnts in NMJ development [57], particularly presynaptic differentiation.

The NMJ is thought to be a highly adaptable structure that can change its form and function. In mature muscles, the levels of physical activity are the primary factors that influence the function of the NMJ [25,31,58]. Traditionally, it was believed that the activation patterns of the skeletal muscle mediated by motor neurons were the primary drivers of NMJ plasticity and the resulting determination of the muscle fiber type. In other words, the ways in which the muscles are used and exercised can impact the structure and function of the NMJs that connect them to the motor neurons. This plasticity allows the NMJ to adapt to different types of muscle use and maintain efficient communication between neurons and muscles [15,59]. Understanding the mechanisms of NMJ plasticity and how it is influenced by physical activity could help researchers to better understand muscle function and develop therapies for muscle-related conditions.

**Figure 1:**
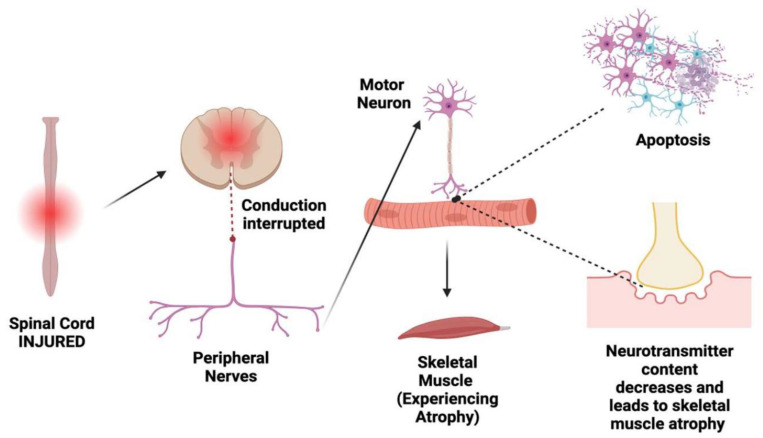
Relation of spinal cord injury to NMJ. Once a spinal cord experiences an injury, its signal conduction to the peripheral nerves is interrupted. Therefore, the motor neurons at the endplate experience cell death (apoptosis), the retransmitted content decreases, and, as a result, the skeletal muscle experiences atrophy. This sequence, starting with SCI, demonstrates how impactful the failure of nerve conduction can be in muscular function. Thus, SCI creates a non-functional NMJ that is hostile to motor neurons.

SCI inflicts structural damage on the NMJ, disrupting the intricate network of branching nerve terminals and compromising the communication between the CNS and skeletal muscles (Figure 1). Axonal damage resulting from SCI hampers the transmission of signals, leading to a cascade of inflammatory responses that further exacerbate the NMJ’s structural integrity [60]. The resulting disruptions contribute to muscle atrophy and functional impairments, underscoring the critical importance of understanding the specific morphological consequences of SCI for the NMJ [5,61]. The accurate measurement and assessment of NMJ alterations post-SCI are essential in unraveling the complexities of the structural damage and developing targeted therapeutic interventions.

A lack of “survival of motor neuron” (SMN) protein stops the growth of the NMJ after birth [62], causing problems in the development of ACh receptor clusters into their proper shape. There are also problems in the structure of the presynaptic area, including poorly developed terminal branches and clumps of intermediate filaments. These structural issues lead to problems in the NMJ’s function, including difficulty transmitting signals between neurons [63]. These problems can be used as indicators of NMJ dysfunction and the overall disease.

## 3. NMJ-Related Disorders

**Figure 2:**
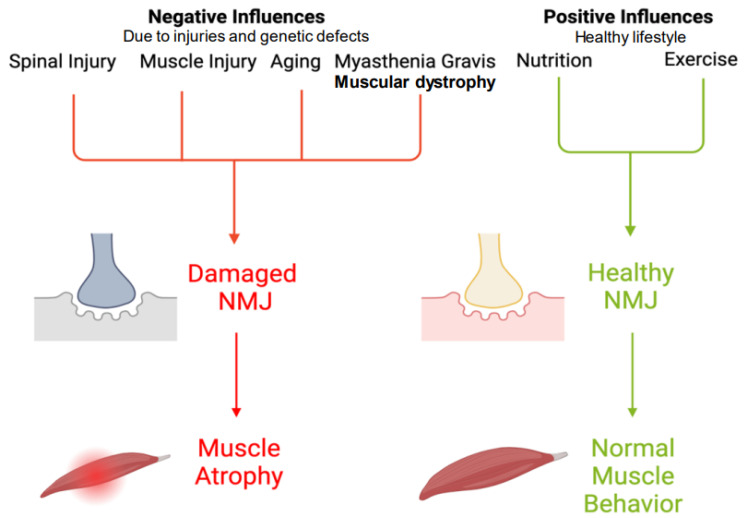
Negative and positive influences on NMJ. SCI as well as other neurodegenerative diseases (e.g., muscle injury, aging, and disorders such as muscular dystrophy and myasthenia gravis) facilitate damage to the NMJ, which therefore induces muscle atrophy. Nutrition and exercise are proactive measures to take that can avoid harming the NMJ. However, nutrition and exercise may not stop an SCI from harming the NMJ, but they may decrease the severity.

Disorders at the NMJ can occur when there is damage, malfunction, or the absence of proteins necessary for the transmission of signals between nerves and muscles [3,64,65]. There is a variety of factors that can disrupt the signaling process between the nerve and muscle at the NMJ. These include the presence of antibodies, CNS injuries, genetic mutations, and certain medications or toxins. When the number or function of the essential proteins responsible for this signaling is impaired, it can also lead to the development of autoimmune disorders. These acquired disorders of the NMJ are the most common type and are the focus of this work. The symptoms of NMJ disorders can often be managed with various treatments, including medications that suppress the immune system or alter its activity [25,64]. Researchers are actively working to improve our understanding of the underlying causes of these conditions, identify new targets for treatment, and develop more specific therapies. NMJ disorders typically decrease nerve cell activity and cause muscle weakness. While the main function of the NMJ is the transmission of signals between the motor neuron and the skeletal muscle fiber to induce muscle contraction and movement, there are also positive and negative influences on the NMJ, as shown in Figure 2.

Two common conditions in clinical practice that involve problems with the transmission of signals between nerves and muscles are MG and Lambert–Eaton myasthenic syndrome (LEMS) [21,66,67]. These disorders often involve defects in neuromuscular transmission. MG is an autoimmune disorder in which muscle weakness occurs because of the impairment of the NMJ and neuromuscular transmission [21,68,69]. The most common symptoms are weakness of the eye muscles and of the muscles that control breathing and swallowing. The disease can also affect the power of the neck, arms, legs, and torso [70,71,72]. An abnormal reaction to an error in the transmission between nerve impulses and muscles causes MG. It is a generalized disorder that manifests initially as weakness and usually as eye muscle weakness. Other myasthenic gravis can be seen in limb-girdle weakness and respiratory and throat muscles, which can be life-threatening exacerbations of MG when breathing or swallowing. The pathways targeted in MG may be related to the stability of the endplate and postsynaptic membrane [73,74,75]. This stability is controlled by ACh clustering and having an unnecessary amount of ACh [76,77]; however, there is the possibility that the NMJ will be destabilized. Autoimmune antibodies directed against the voltage-gated calcium channels in the presynaptic membranes of motor nerve terminals cause reduced neuromuscular transmission [78], resulting in characteristic muscle weakness in patients with LEMS. These channels play a key role in transmitting signals between nerves and muscles, and their dysfunction or destruction can lead to clinical symptoms [67,79].

These are not the only disorders that may occur, but they are the most common. There are also others, such as congenital myasthenic syndromes, a group of inherited disorders that affect the transmission of nerve impulses at the NMJ [15,16,80], causing weakness in voluntary muscles. Botulism is a bacterial infection that also produces toxins and disrupts the transmission of nerve impulses at the NMJ [81], resulting in weakness in voluntary muscles. Additionally, neuromuscular blocking agents are medications that block the transmission of nerve impulses at the NMJ [82], leading to paralysis in voluntary muscles.

## 4. NMJ and Spinal Cord

At the NMJ, a connection is formed between the nerve terminal of a spinal cord motor neuron and a skeletal muscle (Figure 1). This junction is also surrounded by perisynaptic Schwann cells [83,84,85]. Each muscle fiber has a single NMJ innervated by one motor nerve terminal [86]. The lower motor neurons in the spinal cord directly send monosynaptic input to the muscle fibers in the skeletal muscles. This results in the motor neuron axons, which originate from the spinal cord, having to travel a long distance to reach the muscle fibers. In most skeletal muscles, each muscle fiber is connected to one NMJ [17,86].

**Figure 3:**
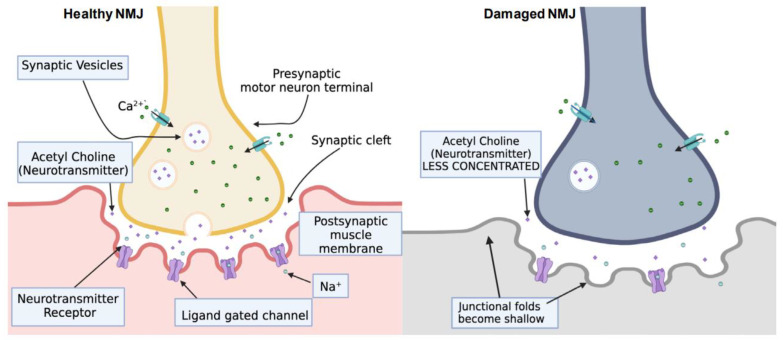
Healthy and functional NMJ compared to damaged NMJ due to SCI. Two enzymes (acetylcholinesterase and choline acetylase) play vital roles in maintaining NMJ integrity. A healthy NMJ has high neurotransmitter concentrations and regular junctional folds with neurotransmitter receptors. A damaged NMJ has limited concentrations of neurotransmitters and the junctional folds become shallow. After SCI, the motor neurons experience endplate death (apoptosis), making the NMJ damaged and dysfunctional in muscle use.

The spinal cord and brainstem largely manage locomotion in vertebrates [87]. Kaufman et al. reported that the brainstem plays a major role in regulating these networks, but most pattern and rhythm generation related to movement occurs within the spinal cord [10]. This study sought to determine whether a segment of the rat spinal cord, when intact, could form functional connections (NMJs) with artificially created 3D muscle tissue. The NMJ’s formation is caused by communication between motor neurons and skeletal muscle fibers [52,88,89,90]. Shortly after they make contact, the motor neurons send signals and release ACh into the space between them (Figure 3). Ultimately, this process shows that the NMJ’s ability to function is closely tied to signals from the spinal cord [10,32].

In a simplified example, it is easy to see how signals travel from the spinal cord to the muscle via this junction. The motor neuron sends an alert to the muscle, instructing it to contract. This signal is carried through the release of a chemical called ACh, released by the motor neuron and picked up by receptors on the muscle fiber [91,92]. Muscle contraction is necessary for movement, and the motor neurons control this process. When a person wishes to move their arm, for example, their brain sends a signal down the spinal cord to the motor neurons in their arm. These motor neurons then release ACh, which triggers the contraction of the muscle fibers in the arm. This results in the movement of the arm. In addition to controlling movement, motor neurons also play a role in reflexes [93,94]. Reflexes are automatic responses to stimuli, such as pulling one’s hand away from a hot stove. When a stimulus is received, a signal is sent from the sensory neurons to the spinal cord, where it is then relayed to the motor neurons [95]. These motor neurons then release ACh, which causes the muscle to contract, resulting in the reflex response (Figure 3). Overall, the motor neurons play a crucial role in the communication between the nervous system and the muscles, allowing for voluntary and reflexive movement.

## 5. SCI and the NMJ

Understanding the injuries (rather than merely the disorders) that may occur is essential to prevent major accidents that could ultimately compromise human movement. One of the critical features of the NMJ is its high level of plasticity, or its ability to adapt and change. The strength of the connection between the motor neuron and the muscle fiber can be modified based on various factors, such as the activity level and certain neurotransmitters’ presence [96,97,98]. This plasticity is essential in fine tuning movement and allows for muscle strength and control changes. Another critical aspect of the NMJ is its role in maintaining muscle tone, or the level of tension in the muscle [17,99]. This is important in maintaining posture and balance and also plays a role in the reflexes that help to protect the body from injury. Dysfunction of the NMJ can lead to muscle weakness or spasticity, affecting movement and mobility [32,99]. The junction is a complex and essential component of the nervous system (Figure 3), enabling communication between the brain and the muscles and allowing for movement and reflexes. Understanding the function and plasticity of this junction can provide insights into various neurological conditions and injuries and help to develop treatments for muscle dysfunction.

SCI may interfere with the brain’s ability to communicate with the rest of the body, causing problems with movement and control. Electrical stimulation therapy can help by using electricity to stimulate the nerves and muscles, allowing for rehabilitation and possibly preventing or managing the medical issues that can occur after a SCI [100,101]. While SCI is somewhat uncommon, it can have serious physical, emotional, and social consequences. In most cases, people do not fully recover from SCI [102]. Each year, approximately 5–10 people out of every million experience SCI [103]. This type of injury involves damage to the nerves, muscles, and tissues surrounding the spinal cord. The extent of the damage can vary, but it can lead to the degeneration of axons and myelin, resulting in impaired neurons and weakened or atrophied skeletal muscles [27,104]. In a study with SCI in rats, Ollivier-Lanvin et al. hypothesized that the NMJ would be compromised after SCI [105]. One key question that this study raises is whether the NMJs may malfunction following human SCI. The study’s findings suggest that NMJ transmission problems may occur after SCI in humans, although the impact on humans may be less severe than observed in adult rats [105].

Neurogenic bowel is a condition that often follows an SCI and occurs after the remodeling of the NMJ [106,107]. The symptoms of the condition are fecal incontinence and constipation. Bowel symptoms occur primarily in patients with a neurological disease history [108,109]. White et al. explored the distal colons of rats after SCI and measured the excitatory junction potentials (EJPs) and nitrergic-mediated slow inhibitory junction potentials (IJPs) [106,110]. These are defined as the depolarization of the muscle after nerve stimulation [111]. The conclusion states that a reduction in EJP and IJP in neuromuscular transmission may cause neurogenic bowel after an SCI—meaning that the “loss-of-function” of the NMJ changes the muscular pathways for the colon.

Without NMJ signaling, the junction typically decreases nerve cell activity, causing muscle weakness and withering [27,32]. As stated, the NMJ is not a permanently fixed structure and has a high degree of plasticity. The morphology can change after exercise, inactivity, aging, and injury [76]. Specifically, in terms of aging and muscle atrophy, muscle quality diminishes over time. Many age-associated pathological changes occur in the NMJ [88,112], and there are strong correlations between axonal transport, the strength of the action potentials, and age. Animal and human experiments have proven that, with age, the presynaptic structure completely changes in terms of axonal denervation, remodeling, and altered branching [56,113]. Overall, whether changes in the NMJ depend strictly on the muscle is not fully understood. However, it is somewhat clear that there is a gradual deterioration in NMJ function with age.

Motor neuron diseases almost always relate to the degeneration of the upper and lower neurons [114,115]. In spinal muscular atrophy (SMA) conditions, many factors may be incorporated into its development. For instance, a neuromuscular gene mutation affects SMA, called the survival motor neuron one gene (SMN1) [76,116]. Studies with mouse models of SMA have shown NMJ abnormalities [117,118]. These studies reported that the loss of SMN in mice with an already fully mature NMJ only changed with injury or age. However, after the loss of SMN in mice without the mature NMJ, there were major phenotypic SMA-like changes [119]. Overall, many more changes may affect the NMJ or vice versa. The NMJ is the central “node” for nerve and muscle transmission, making it a significant component in the muscular function machinery (Figure 2 and Figure 3). However, some factors may reverse and/or have potentially significant effects in causing NMJ dysfunction, such as exercise, nutrition, and trophic factors.

## 6. Possible Therapies for NMJ Dysfunction

Scientists are studying methods to use gene therapy to improve the effects of different types of muscular dystrophy. Researchers hope that by continuing to develop and refine gene and cell therapy techniques, they can effectively treat muscle disorders and ultimately improve the structure and function of the NMJ [120,121,122,123]. Essentially, NMJ fragmentation/denervation occurs in conjunction with decreased skeletal muscle in aging patients (Figure 2). However, these have been stabilized via transgenic expression—essentially, gene therapy. This has been able to improve the ACh receptors [124]. Moreover, interventions in mouse models to help to stabilize the NMJ have not proven to be fully effective and rather delay the NMJ decline modestly. Gene therapy can ameliorate the symptoms but is continuing to be investigated.

A habit change can also alter the NMJ physiology and help to prevent the development of NMJ-related disorders—the most significant one being caloric restriction (reducing one’s daily average caloric intake). This may be successful because of the relationship between caloric restriction and aging. There are correlations between decreasing caloric intake and reduced physical aging in muscle and tissue [125]. Research has found that caloric restriction in mice can help to postpone the emergence of diseases associated with aging, including the deterioration of motor neurons and modifications at the NMJ. Studies of mice aged up to 24 months old have shown that caloric restriction leads to the significant preservation of the NMJ, with fewer instances of damage to the postsynaptic NMJ and axonal degeneration compared to mice that were the same age but did not undergo caloric restriction [126,127,128]. It seems clear that there are many ways to target the stability of the NMJ to improve muscle function or slow muscle decline. However, it is important to note that the effectiveness of different molecular targets may vary depending on whether they directly impact the NMJ’s stability or whether they work indirectly through improving muscle structure and function or reducing muscle degeneration.

Studies suggest that autophagy interacts with secretory pathways to regulate axonal homeostasis and neurotransmission at the NMJ [129,130,131,132,133]. Autophagy can be activated both under short and prolonged stress and is required to clear the cell of dysfunctional organelles and altered proteins. The inhibition of autophagy in muscle is known to have a major impact on neuromuscular synaptic function and muscle strength, which may affect the lifespans of animals. The inhibition of autophagy also exacerbates aging phenotypes in muscle, such as mitochondrial dysfunction, oxidative stress, and profound weakness. These effects of autophagy may contribute to the regulation of neurotransmission, likely at presynaptic sites, but there is minimal information on such effects at this time. The inhibition of autophagy has also been shown to accumulate vesicles close to the endplates, which impairs AChR turnover, suggesting that the involvement of autophagy is important in NMJ maintenance. In this scenario, glial cells can play a major role in the formation and maintenance of the neuromuscular junction [134].

A recent study suggests that the maintenance of a normal autophagy level in the skeletal muscle can positively affect whole-body metabolism and NMJ preservation [135]. In particular, mounting evidence demonstrates that, in motor neuron diseases such as ALS, SBMA, and SMA, alterations within the NMJ occur early during disease progression [48,136,137,138,139]. The degeneration of the distal axon and NMJ is considered a key and early feature of the pathology that accompanies motor neuron loss in people with ALS [137]. The Notch pathway plays an important role in glial activation and alterations of motor neurons [140]. It would be interesting to examine whether SCI influences the Notch pathway regarding the alteration of the NMJ. A recent study suggests that SBMA causes the neuromuscular synapses to become weak and muscles to become hyperexcitable [48]. This study has shown that SBMA is accompanied by marked defects in neuromuscular synaptic transmission involving both presynaptic and postsynaptic mechanisms. Studies also suggest that muscle fiber atrophy and weakness may not simply be the result of lower motor neuron degeneration, but instead that the muscle fibers may be the sites of crucial pathogenic events in these diseases [136]. Overall, NMJ repair by modulating autophagy and other detrimental factors could be an appropriate therapy for muscle disorders in chronic SCI and motor neuron diseases.

## 7. Conclusions

The connection between the nerve endings of spinal cord motor neurons and skeletal muscles is known as the NMJ. The physical layout of the NMJ determines where the molecules responsible for transmitting signals operate. This means that the NMJ’s structural elements significantly affect signal transmission’s effectiveness. Recently, scientists have started to pay more attention to the role of the NMJ in developing weakness caused by muscle injury, aging, and muscle disease. This is a change from the traditional focus on the muscle itself. However, it is becoming clear that multiple factors are involved in weakening the neuromuscular system, including changes in the motor neurons and muscle fibers, as well as other cell types and factors. The complex relationship between muscle health, nerve health, and the NMJ’s structure and function is an ongoing area of research, particularly in the context of aging and muscle disease, and these areas should be investigated further for the development of new therapies for NMJ disorders.

SCI not only results in axonal damage but also induces a cascade of inflammatory responses, compromising the NMJ’s structural integrity and contributing to muscle atrophy. The interplay between SCI and NMJ disorders poses challenges to motor function and muscle strength and causes long-term disability. Recognizing the NMJ’s central role in transmitting signals from the spinal cord to the muscle fibers underscores the necessity of studying SCI alongside NMJ disorders. The dynamic nature of the NMJ, subject to changes in response to physical activity, aging, and injury, adds complexity to the understanding of its structure and function. Further studies focusing on autophagy in SCI and motor neuron diseases may reveal insights into the formation and function of the NMJ and the alteration of the NMJ’s structure by SCI.

As we move forward, a comprehensive understanding of the NMJ’s plasticity, its response to interventions, and its intricate relationship with SCI will pave the way for innovative treatments. Unraveling the molecular intricacies governing NMJ stability holds potential not only in addressing specific disorders but also in enhancing overall muscle function and mobility. Future research may explore advanced imaging techniques, precision medicine approaches, and targeted therapeutics, offering new avenues to improve the lives of those affected by NMJ-related complications and SCI.

## References

[1] Jones R.A., Harrison C., Eaton S.L., Llavero Hurtado M., Graham L.C., Alkhammash L., Oladiran O.A., Gale A., Lamont D.J., Simpson H. (2017). Cellular and Molecular Anatomy of the Human Neuromuscular Junction. Cell Rep..

[2] Jimsheleishvili S., Marwaha K., Sherman A.L. (2023). Physiology, Neuromuscular Transmission. StatPearls.

[3] Hill M. (2003). The neuromuscular junction disorders. J. Neurol. Neurosurg. Psychiatry.

[4] Lee M., Kiernan M.C., Macefield V.G., Lee B.B., Lin C.S. (2015). Short-term peripheral nerve stimulation ameliorates axonal dysfunction after spinal cord injury. J. Neurophysiol..

[5] Xu X., Talifu Z., Zhang C.J., Gao F., Ke H., Pan Y.Z., Gong H., Du H.Y., Yu Y., Jing Y.L. (2023). Mechanism of skeletal muscle atrophy after spinal cord injury: A narrative review. Front. Nutr..

[6] Egawa N., Lok J., Washida K., Arai K. (2017). Mechanisms of Axonal Damage and Repair after Central Nervous System Injury. Transl. Stroke Res..

[7] Alizadeh A., Dyck S.M., Karimi-Abdolrezaee S. (2019). Traumatic Spinal Cord Injury: An Overview of Pathophysiology, Models and Acute Injury Mechanisms. Front. Neurol..

[8] Burns A.S., Jawaid S., Zhong H., Yoshihara H., Bhagat S., Murray M., Roy R.R., Tessler A., Son Y.J. (2007). Paralysis elicited by spinal cord injury evokes selective disassembly of neuromuscular synapses with and without terminal sprouting in ankle flexors of the adult rat. J. Comp. Neurol..

[9] Thomas C.K., Bakels R., Klein C.S., Zijdewind I. (2014). Human spinal cord injury: Motor unit properties and behaviour. Acta Physiol..

[10] Kaufman C.D., Liu S.C., Cvetkovic C., Lee C.A., Naseri Kouzehgarani G., Gillette R., Bashir R., Gillette M.U. (2020). Emergence of functional neuromuscular junctions in an engineered, multicellular spinal cord-muscle bioactuator. APL Bioeng..

[11] Omar A., Marwaha K., Bollu P.C. (2023). Physiology, Neuromuscular Junction. StatPearls.

[12] Liu W., Chakkalakal J.V. (2018). The Composition, Development, and Regeneration of Neuromuscular Junctions. Curr. Top. Dev. Biol..

[13] Sanes J.R., Lichtman J.W. (1999). Development of the vertebrate neuromuscular junction. Annu. Rev. Neurosci..

[14] Sanes J.N. (2003). Neocortical mechanisms in motor learning. Curr. Opin. Neurobiol..

[15] Rodriguez Cruz P.M., Cossins J., Beeson D., Vincent A. (2020). The Neuromuscular Junction in Health and Disease: Molecular Mechanisms Governing Synaptic Formation and Homeostasis. Front. Mol. Neurosci..

[16] Zou S., Pan B.X. (2022). Post-synaptic specialization of the neuromuscular junction: Junctional folds formation, function, and disorders. Cell Biosci..

[17] Tintignac L.A., Brenner H.R., Ruegg M.A. (2015). Mechanisms Regulating Neuromuscular Junction Development and Function and Causes of Muscle Wasting. Physiol. Rev..

[18] Molotsky E., Liu Y., Lieberman A.P., Merry D.E. (2022). Neuromuscular junction pathology is correlated with differential motor unit vulnerability in spinal and bulbar muscular atrophy. Acta Neuropathol. Commun..

[19] Kotaich F., Caillol D., Bomont P. (2023). Neurofilaments in health and Charcot-Marie-Tooth disease. Front. Cell Dev. Biol..

[20] King P.H. (2024). Skeletal muscle as a molecular and cellular biomarker of disease progression in amyotrophic lateral sclerosis: A narrative review. Neural Regen. Res..

[21] Iorio R. (2024). Myasthenia gravis: The changing treatment landscape in the era of molecular therapies. Nat. Rev. Neurol..

[22] Mishra A.K., Varma A. (2023). Myasthenia Gravis: A Systematic Review. Cureus.

[23] Gilhus N.E., Tzartos S., Evoli A., Palace J., Burns T.M., Verschuuren J. (2019). Myasthenia gravis. Nat. Rev. Dis. Primers.

[24] Sleigh J.N., Burgess R.W., Gillingwater T.H., Cader M.Z. (2014). Morphological analysis of neuromuscular junction development and degeneration in rodent lumbrical muscles. J. Neurosci. Methods.

[25] Ham D.J., Borsch A., Lin S., Thurkauf M., Weihrauch M., Reinhard J.R., Delezie J., Battilana F., Wang X., Kaiser M.S. (2020). The neuromuscular junction is a focal point of mTORC1 signaling in sarcopenia. Nat. Commun..

[26] Tran A.P., Warren P.M., Silver J. (2018). The Biology of Regeneration Failure and Success After Spinal Cord Injury. Physiol. Rev..

[27] Myatich A., Haque A., Sole C., Banik N.L. (2023). Clemastine in remyelination and protection of neurons and skeletal muscle after spinal cord injury. Neural Regen. Res..

[28] Lou W.P., Mateos A., Koch M., Klussman S., Yang C., Lu N., Kumar S., Limpert S., Gopferich M., Zschaetzsch M. (2017). Regulation of Adult CNS Axonal Regeneration by the Post-transcriptional Regulator Cpeb1. Front. Mol. Neurosci..

[29] Li L.K., Huang W.C., Hsueh Y.Y., Yamauchi K., Olivares N., Davila R., Fang J., Ding X., Zhao W., Soto J. (2022). Intramuscular delivery of neural crest stem cell spheroids enhances neuromuscular regeneration after denervation injury. Stem Cell Res. Ther..

[30] Otzel D.M., Lee J., Ye F., Borst S.E., Yarrow J.F. (2018). Activity-Based Physical Rehabilitation with Adjuvant Testosterone to Promote Neuromuscular Recovery after Spinal Cord Injury. Int. J. Mol. Sci..

[31] Gazzola M., Martinat C. (2023). Unlocking the Complexity of Neuromuscular Diseases: Insights from Human Pluripotent Stem Cell-Derived Neuromuscular Junctions. Int. J. Mol. Sci..

[32] Glowacka A., Ji B., Szczepankiewicz A.A., Skup M., Gajewska-Wozniak O. (2022). BDNF Spinal Overexpression after Spinal Cord Injury Partially Protects Soleus Neuromuscular Junction from Disintegration, Increasing VAChT and AChE Transcripts in Soleus but Not Tibialis Anterior Motoneurons. Biomedicines.

[33] Slater C.R. (2017). The Structure of Human Neuromuscular Junctions: Some Unanswered Molecular Questions. Int. J. Mol. Sci..

[34] Alijevic O., Jaka O., Alzualde A., Maradze D., Xia W., Frentzel S., Gifford A.N., Peitsch M.C., Hoeng J., Koshibu K. (2022). Differentiating the Neuropharmacological Properties of Nicotinic Acetylcholine Receptor-Activating Alkaloids. Front. Pharmacol..

[35] Rudolf R., Khan M.M., Witzemann V. (2019). Motor Endplate-Anatomical, Functional, and Molecular Concepts in the Historical Perspective. Cells.

[36] Reichardt L.F., Kelly R.B. (1983). A molecular description of nerve terminal function. Annu. Rev. Biochem..

[37] Williamson J.M., Lyons D.A. (2018). Myelin Dynamics Throughout Life: An Ever-Changing Landscape?. Front. Cell. Neurosci..

[38] Liu B., Xin W., Tan J.R., Zhu R.P., Li T., Wang D., Kan S.S., Xiong D.K., Li H.H., Zhang M.M. (2019). Myelin sheath structure and regeneration in peripheral nerve injury repair. Proc. Natl. Acad. Sci. USA.

[39] Siksou L., Rostaing P., Lechaire J.P., Boudier T., Ohtsuka T., Fejtova A., Kao H.T., Greengard P., Gundelfinger E.D., Triller A. (2007). Three-dimensional architecture of presynaptic terminal cytomatrix. J. Neurosci..

[40] Lucic V., Yang T., Schweikert G., Forster F., Baumeister W. (2005). Morphological characterization of molecular complexes present in the synaptic cleft. Structure.

[41] Rusakov D.A., Savtchenko L.P., Zheng K., Henley J.M. (2011). Shaping the synaptic signal: Molecular mobility inside and outside the cleft. Trends Neurosci..

[42] Zhu Y., Warrenfelt C.I.C., Flannery J.C., Lindgren C.A. (2021). Extracellular Protons Mediate Presynaptic Homeostatic Potentiation at the Mouse Neuromuscular Junction. Neuroscience.

[43] Cijsouw T., Ramsey A.M., Lam T.T., Carbone B.E., Blanpied T.A., Biederer T. (2018). Mapping the Proteome of the Synaptic Cleft through Proximity Labeling Reveals New Cleft Proteins. Proteomes.

[44] Ho T.N.T., Abraham N., Lewis R.J. (2020). Structure-Function of Neuronal Nicotinic Acetylcholine Receptor Inhibitors Derived from Natural Toxins. Front. Neurosci..

[45] Miyazawa A., Fujiyoshi Y., Unwin N. (2003). Structure and gating mechanism of the acetylcholine receptor pore. Nature.

[46] Day N.C., Wood S.J., Ince P.G., Volsen S.G., Smith W., Slater C.R., Shaw P.J. (1997). Differential localization of voltage-dependent calcium channel alpha1 subunits at the human and rat neuromuscular junction. J. Neurosci..

[47] Wood S.J., Slater C.R. (1997). The contribution of postsynaptic folds to the safety factor for neuromuscular transmission in rat fast- and slow-twitch muscles. J. Physiol..

[48] Xu Y., Halievski K., Henley C., Atchison W.D., Katsuno M., Adachi H., Sobue G., Breedlove S.M., Jordan C.L. (2016). Defects in Neuromuscular Transmission May Underlie Motor Dysfunction in Spinal and Bulbar Muscular Atrophy. J. Neurosci..

[49] Fulceri F., Biagioni F., Limanaqi F., Busceti C.L., Ryskalin L., Lenzi P., Fornai F. (2021). Ultrastructural characterization of peripheral denervation in a mouse model of Type III spinal muscular atrophy. J. Neural Transm..

[50] Verma S., Khurana S., Vats A., Sahu B., Ganguly N.K., Chakraborti P., Gourie-Devi M., Taneja V. (2022). Neuromuscular Junction Dysfunction in Amyotrophic Lateral Sclerosis. Mol. Neurobiol..

[51] Cipriani S., Phan V., Medard J.J., Horvath R., Lochmuller H., Chrast R., Roos A., Spendiff S. (2018). Neuromuscular Junction Changes in a Mouse Model of Charcot-Marie-Tooth Disease Type 4C. Int. J. Mol. Sci..

[52] Davis L.A., Fogarty M.J., Brown A., Sieck G.C. (2022). Structure and Function of the Mammalian Neuromuscular Junction. Compr. Physiol..

[53] Mejia Maza A., Jarvis S., Lee W.C., Cunningham T.J., Schiavo G., Secrier M., Fratta P., Sleigh J.N., Fisher E.M.C., Sudre C.H. (2021). NMJ-Analyser identifies subtle early changes in mouse models of neuromuscular disease. Sci. Rep..

[54] Lin W., McArdle J.J. (2021). The NMJ as a model synapse: New perspectives on synapse formation, function, and maintenance. Neurosci. Lett..

[55] Alvarez-Suarez P., Gawor M., Proszynski T.J. (2020). Perisynaptic schwann cells—The multitasking cells at the developing neuromuscular junctions. Semin. Cell Dev. Biol..

[56] Natarajan A., Sethumadhavan A., Krishnan U.M. (2019). Toward Building the Neuromuscular Junction: In Vitro Models to Study Synaptogenesis and Neurodegeneration. ACS Omega.

[57] Shen C., Li L., Zhao K., Bai L., Wang A., Shu X., Xiao Y., Zhang J., Zhang K., Hui T. (2018). Motoneuron Wnts regulate neuromuscular junction development. eLife.

[58] Arnold A.S., Gill J., Christe M., Ruiz R., McGuirk S., St-Pierre J., Tabares L., Handschin C. (2014). Morphological and functional remodelling of the neuromuscular junction by skeletal muscle PGC-1alpha. Nat. Commun..

[59] Wang X., Rich M.M. (2018). Homeostatic synaptic plasticity at the neuromuscular junction in myasthenia gravis. Ann. N. Y. Acad. Sci..

[60] Yin L., Li N., Jia W., Wang N., Liang M., Yang X., Du G. (2021). Skeletal muscle atrophy: From mechanisms to treatments. Pharmacol. Res..

[61] Lee Y.I., Mikesh M., Smith I., Rimer M., Thompson W. (2011). Muscles in a mouse model of spinal muscular atrophy show profound defects in neuromuscular development even in the absence of failure in neuromuscular transmission or loss of motor neurons. Dev. Biol..

[62] Singh R.N., Howell M.D., Ottesen E.W., Singh N.N. (2017). Diverse role of survival motor neuron protein. Biochim. Biophys. Acta Gene Regul. Mech..

[63] Kariya S., Park G.H., Maeno-Hikichi Y., Leykekhman O., Lutz C., Arkovitz M.S., Landmesser L.T., Monani U.R. (2008). Reduced SMN protein impairs maturation of the neuromuscular junctions in mouse models of spinal muscular atrophy. Hum. Mol. Genet..

[64] Verschuuren J., Strijbos E., Vincent A. (2016). Neuromuscular junction disorders. Handb. Clin. Neurol..

[65] Pasnoor M., Dimachkie M.M. (2019). Approach to Muscle and Neuromuscular Junction Disorders. Continuum.

[66] Kesner V.G., Oh S.J., Dimachkie M.M., Barohn R.J. (2018). Lambert-Eaton Myasthenic Syndrome. Neurol. Clin..

[67] Hulsbrink R., Hashemolhosseini S. (2014). Lambert-Eaton myasthenic syndrome—Diagnosis, pathogenesis and therapy. Clin. Neurophysiol..

[68] Carr A.S., Cardwell C.R., McCarron P.O., McConville J. (2010). A systematic review of population based epidemiological studies in Myasthenia Gravis. BMC Neurol..

[69] Takamori M. (2020). Myasthenia Gravis: From the Viewpoint of Pathogenicity Focusing on Acetylcholine Receptor Clustering, Trans-Synaptic Homeostasis and Synaptic Stability. Front. Mol. Neurosci..

[70] Dresser L., Wlodarski R., Rezania K., Soliven B. (2021). Myasthenia Gravis: Epidemiology, Pathophysiology and Clinical Manifestations. J. Clin. Med..

[71] Phillips W.D., Vincent A. (2016). Pathogenesis of myasthenia gravis: Update on disease types, models, and mechanisms. F1000Research.

[72] Koneczny I., Herbst R. (2019). Myasthenia Gravis: Pathogenic Effects of Autoantibodies on Neuromuscular Architecture. Cells.

[73] Nagaoka A., Tsujino A., Shiraishi H., Kanamoto T., Shima T., Yoshimura S., Miyazaki T., Tateishi Y., Tsujihata M., Motomura M. (2022). Motor end-plate analysis to diagnose immune-mediated myasthenia gravis in seronegative patients. J. Neurol. Sci..

[74] Vilquin J.T., Bayer A.C., Le Panse R., Berrih-Aknin S. (2019). The Muscle Is Not a Passive Target in Myasthenia Gravis. Front. Neurol..

[75] Richman D.P. (2015). The Future of Research in Myasthenia. JAMA Neurol..

[76] Iyer C.C., Chugh D., Bobbili P.J., Iii A.J.B., Crum A.E., Yi A.F., Kaspar B.K., Meyer K.C., Burghes A.H.M., Arnold W.D. (2021). Follistatin-induced muscle hypertrophy in aged mice improves neuromuscular junction innervation and function. Neurobiol. Aging.

[77] Barrantes F.J. (2021). Possible implications of dysregulated nicotinic acetylcholine receptor diffusion and nanocluster formation in myasthenia gravis. Neural Regen. Res..

[78] Bekircan-Kurt C.E., Derle Ciftci E., Kurne A.T., Anlar B. (2015). Voltage gated calcium channel antibody-related neurological diseases. World J. Clin. Cases.

[79] Totzeck A., Mummel P., Kastrup O., Hagenacker T. (2016). Clinical Features of Neuromuscular Disorders in Patients with N-Type Voltage-Gated Calcium Channel Antibodies. Eur. J. Transl. Myol..

[80] Nicole S., Azuma Y., Bauche S., Eymard B., Lochmuller H., Slater C. (2017). Congenital Myasthenic Syndromes or Inherited Disorders of Neuromuscular Transmission: Recent Discoveries and Open Questions. J. Neuromuscul. Dis..

[81] Machamer J.B., Vazquez-Cintron E.J., Stenslik M.J., Pagarigan K.T., Bradford A.B., Ondeck C.A., McNutt P.M. (2023). Neuromuscular recovery from botulism involves multiple forms of compensatory plasticity. Front. Cell. Neurosci..

[82] Sellin L.C. (1981). The action of batulinum toxin at the neuromuscular junction. Med. Biol..

[83] Balice-Gordon R.J. (1996). Dynamic roles at the neuromuscular junction. Schwann cells. Curr. Biol. CB.

[84] Ko C.P., Robitaille R. (2015). Perisynaptic Schwann Cells at the Neuromuscular Synapse: Adaptable, Multitasking Glial Cells. Cold Spring Harb. Perspect. Biol..

[85] Alhindi A., Boehm I., Forsythe R.O., Miller J., Skipworth R.J.E., Simpson H., Jones R.A., Gillingwater T.H. (2021). Terminal Schwann cells at the human neuromuscular junction. Brain Commun..

[86] Nishimune H., Shigemoto K. (2018). Practical Anatomy of the Neuromuscular Junction in Health and Disease. Neurol. Clin..

[87] Basinger H., Hogg J.P. (2023). Neuroanatomy, Brainstem. StatPearls.

[88] Iyer S.R., Shah S.B., Lovering R.M. (2021). The Neuromuscular Junction: Roles in Aging and Neuromuscular Disease. Int. J. Mol. Sci..

[89] Zelada D., Bermedo-Garcia F., Collao N., Henriquez J.P. (2021). Motor function recovery: Deciphering a regenerative niche at the neuromuscular synapse. Biol. Rev. Camb. Philos. Soc..

[90] Alavi-Moghadam S., Kokabi-Hamidpour S., Rezaei-Tavirani M., Larijani B., Arjmand R., Rahim F., Rezazadeh-Mafi A., Adibi H., Arjmand B. (2024). Neuromuscular Junction-on-a-Chip for Amyotrophic Lateral Sclerosis Modeling. Methods Mol. Biol..

[91] Rimington R.P., Fleming J.W., Capel A.J., Wheeler P.C., Lewis M.P. (2021). Bioengineered model of the human motor unit with physiologically functional neuromuscular junctions. Sci. Rep..

[92] Sam C., Bordoni B. (2023). Physiology, Acetylcholine. StatPearls.

[93] Masugi Y., Obata H., Inoue D., Kawashima N., Nakazawa K. (2017). Neural effects of muscle stretching on the spinal reflexes in multiple lower-limb muscles. PLoS ONE.

[94] Masugi Y., Sasaki A., Kaneko N., Nakazawa K. (2019). Remote muscle contraction enhances spinal reflexes in multiple lower-limb muscles elicited by transcutaneous spinal cord stimulation. Exp. Brain Res..

[95] Kuo I.Y., Ehrlich B.E. (2015). Signaling in muscle contraction. Cold Spring Harb. Perspect. Biol..

[96] Yue G.H., Clark B.C., Li S., Vaillancourt D.E. (2017). Understanding Neuromuscular System Plasticity to Improve Motor Function in Health, Disease, and Injury. Neural Plast..

[97] Wall E.M., Woolley S.C. (2020). Acetylcholine in action. eLife.

[98] Rima M., Lattouf Y., Abi Younes M., Bullier E., Legendre P., Mangin J.M., Hong E. (2020). Dynamic regulation of the cholinergic system in the spinal central nervous system. Sci. Rep..

[99] Gulino R., Vicario N., Giunta M.A.S., Spoto G., Calabrese G., Vecchio M., Gulisano M., Leanza G., Parenti R. (2019). Neuromuscular Plasticity in a Mouse Neurotoxic Model of Spinal Motoneuronal Loss. Int. J. Mol. Sci..

[100] Ho C., Atchison K., Noonan V.K., McKenzie N., Cadel L., Ganshorn H., Rivera J.M.B., Yousefi C., Guilcher S.J.T. (2021). Models of Care Delivery from Rehabilitation to Community for Spinal Cord Injury: A Scoping Review. J. Neurotrauma.

[101] Ho C.H., Triolo R.J., Elias A.L., Kilgore K.L., DiMarco A.F., Bogie K., Vette A.H., Audu M.L., Kobetic R., Chang S.R. (2014). Functional electrical stimulation and spinal cord injury. Phys. Med. Rehabil. Clin. N. Am..

[102] Chen Y., Tang Y., Vogel L.C., Devivo M.J. (2013). Causes of spinal cord injury. Top. Spinal Cord Inj. Rehabil..

[103] Bennett J., Das J.M., Emmady P.D. (2023). Spinal Cord Injuries. StatPearls.

[104] Ahuja C.S., Nori S., Tetreault L., Wilson J., Kwon B., Harrop J., Choi D., Fehlings M.G. (2017). Traumatic Spinal Cord Injury-Repair and Regeneration. Neurosurgery.

[105] Ollivier-Lanvin K., Lemay M.A., Tessler A., Burns A.S. (2009). Neuromuscular transmission failure and muscle fatigue in ankle muscles of the adult rat after spinal cord injury. J. Appl. Physiol..

[106] Rodriguez G.M., Gater D.R. (2022). Neurogenic Bowel and Management after Spinal Cord Injury: A Narrative Review. J. Pers. Med..

[107] Camilleri M. (2021). Gastrointestinal motility disorders in neurologic disease. J. Clin. Investig..

[108] Emmanuel A. (2019). Neurogenic bowel dysfunction. F1000Research.

[109] Choukou M.A., Best K.L., Craven B.C., Hitzig S.L. (2019). Identifying and Classifying Quality of Life Tools for Assessing Neurogenic Bowel Dysfunction after Spinal Cord Injury. Top. Spinal Cord Inj. Rehabil..

[110] White A.R., Werner C.M., Holmes G.M. (2020). Diminished enteric neuromuscular transmission in the distal colon following experimental spinal cord injury. Exp. Neurol..

[111] Blakeley A.G., Dunn P.M., Petersen S.A. (1989). Properties of excitatory junction potentials and currents in smooth muscle cells of the mouse vas deferens. J. Auton Nerv. Syst..

[112] Khosa S., Trikamji B., Khosa G.S., Khanli H.M., Mishra S.K. (2019). An Overview of Neuromuscular Junction Aging Findings in Human and Animal Studies. Curr. Aging Sci..

[113] Fahim M.A., Robbins N. (1982). Ultrastructural studies of young and old mouse neuromuscular junctions. J. Neurocytol..

[114] Petri S., Grehl T., Grosskreutz J., Hecht M., Hermann A., Jesse S., Lingor P., Loscher W., Maier A., Schoser B. (2023). Guideline “Motor neuron diseases” of the German Society of Neurology (Deutsche Gesellschaft fur Neurologie). Neurol. Res. Pract..

[115] Heinrich F., Cordts I., Gunther R., Stolte B., Zeller D., Schroter C., Weyen U., Regensburger M., Wolf J., Schneider I. (2023). Economic evaluation of Motor Neuron Diseases: A nationwide cross-sectional analysis in Germany. J. Neurol..

[116] Iyer C.C., McGovern V.L., Murray J.D., Gombash S.E., Zaworski P.G., Foust K.D., Janssen P.M., Burghes A.H. (2015). Low levels of Survival Motor Neuron protein are sufficient for normal muscle function in the SMNDelta7 mouse model of SMA. Hum. Mol. Genet..

[117] Murray L.M., Comley L.H., Thomson D., Parkinson N., Talbot K., Gillingwater T.H. (2008). Selective vulnerability of motor neurons and dissociation of pre- and post-synaptic pathology at the neuromuscular junction in mouse models of spinal muscular atrophy. Hum. Mol. Genet..

[118] Feng Z., Lam S., Tenn E.S., Ghosh A.S., Cantor S., Zhang W., Yen P.F., Chen K.S., Burden S., Paushkin S. (2021). Activation of Muscle-Specific Kinase (MuSK) Reduces Neuromuscular Defects in the Delta7 Mouse Model of Spinal Muscular Atrophy (SMA). Int. J. Mol. Sci..

[119] Boido M., Vercelli A. (2016). Neuromuscular Junctions as Key Contributors and Therapeutic Targets in Spinal Muscular Atrophy. Front. Neuroanat..

[120] Ng S.Y., Ljubicic V. (2020). Recent insights into neuromuscular junction biology in Duchenne muscular dystrophy: Impacts, challenges, and opportunities. EBioMedicine.

[121] Jones R.A., Reich C.D., Dissanayake K.N., Kristmundsdottir F., Findlater G.S., Ribchester R.R., Simmen M.W., Gillingwater T.H. (2016). NMJ-morph reveals principal components of synaptic morphology influencing structure-function relationships at the neuromuscular junction. Open Biol..

[122] Lundt S., Zhang N., Wang X., Polo-Parada L., Ding S. (2020). The effect of NAMPT deletion in projection neurons on the function and structure of neuromuscular junction (NMJ) in mice. Sci. Rep..

[123] Stilhano R.S., Martins L., Ingham S.J.M., Pesquero J.B., Huard J. (2015). Gene and cell therapy for muscle regeneration. Curr. Rev. Musculoskelet Med..

[124] Zhao K., Shen C., Li L., Wu H., Xing G., Dong Z., Jing H., Chen W., Zhang H., Tan Z. (2018). Sarcoglycan Alpha Mitigates Neuromuscular Junction Decline in Aged Mice by Stabilizing LRP4. J. Neurosci..

[125] Sohal R.S., Forster M.J. (2014). Caloric restriction and the aging process: A critique. Free. Radic. Biol. Med..

[126] Deschenes M.R. (2011). Motor unit and neuromuscular junction remodeling with aging. Curr. Aging Sci..

[127] Valdez G., Tapia J.C., Kang H., Clemenson G.D., Gage F.H., Lichtman J.W., Sanes J.R. (2010). Attenuation of age-related changes in mouse neuromuscular synapses by caloric restriction and exercise. Proc. Natl. Acad. Sci. USA.

[128] Pratt S.J.P., Valencia A.P., Le G.K., Shah S.B., Lovering R.M. (2015). Pre- and postsynaptic changes in the neuromuscular junction in dystrophic mice. Front. Physiol..

[129] Limanaqi F., Busceti C.L., Biagioni F., Cantini F., Lenzi P., Fornai F. (2020). Cell-Clearing Systems Bridging Repeat Expansion Proteotoxicity and Neuromuscular Junction Alterations in ALS and SBMA. Int. J. Mol. Sci..

[130] Lienard C., Pintart A., Bomont P. (2024). Neuronal Autophagy: Regulations and Implications in Health and Disease. Cells.

[131] Fleming A., Bourdenx M., Fujimaki M., Karabiyik C., Krause G.J., Lopez A., Martin-Segura A., Puri C., Scrivo A., Skidmore J. (2022). The different autophagy degradation pathways and neurodegeneration. Neuron.

[132] Rudnick N.D., Griffey C.J., Guarnieri P., Gerbino V., Wang X., Piersaint J.A., Tapia J.C., Rich M.M., Maniatis T. (2017). Distinct roles for motor neuron autophagy early and late in the SOD1(G93A) mouse model of ALS. Proc. Natl. Acad. Sci. USA.

[133] Sidibe D.K., Kulkarni V.V., Dong A., Herr J.B., Vogel M.C., Stempel M.H., Maday S. (2022). Brain-derived neurotrophic factor stimulates the retrograde pathway for axonal autophagy. J. Biol. Chem..

[134] Feng Z., Ko C.P. (2008). The role of glial cells in the formation and maintenance of the neuromuscular junction. Ann. N. Y. Acad. Sci..

[135] Demontis F., Perrimon N. (2010). FOXO/4E-BP signaling in Drosophila muscles regulates organism-wide proteostasis during aging. Cell.

[136] Dupuis L., Echaniz-Laguna A. (2010). Skeletal muscle in motor neuron diseases: Therapeutic target and delivery route for potential treatments. Curr. Drug Targets.

[137] Clark J.A., Southam K.A., Blizzard C.A., King A.E., Dickson T.C. (2016). Axonal degeneration, distal collateral branching and neuromuscular junction architecture alterations occur prior to symptom onset in the SOD1(G93A) mouse model of amyotrophic lateral sclerosis. J. Chem. Neuroanat..

[138] Onodera K., Shimojo D., Ishihara Y., Yano M., Miya F., Banno H., Kuzumaki N., Ito T., Okada R., de Araujo Herculano B. (2020). Unveiling synapse pathology in spinal bulbar muscular atrophy by genome-wide transcriptome analysis of purified motor neurons derived from disease specific iPSCs. Mol. Brain.

[139] Biagioni F., Ferrucci M., Ryskalin L., Fulceri F., Lazzeri G., Calierno M.T., Busceti C.L., Ruffoli R., Fornai F. (2017). Protective effects of long-term lithium administration in a slowly progressive SMA mouse model. Arch Ital Biol..

[140] Wang S.Y., Ren M., Jiang H.Z., Wang J., Jiang H.Q., Yin X., Qi Y., Wang X.D., Dong G.T., Wang T.H. (2015). Notch pathway is activated in cell culture and mouse models of mutant SOD1-related familial amyotrophic lateral sclerosis, with suppression of its activation as an additional mechanism of neuroprotection for lithium and valproate. Neuroscience.

